# Nature and Revelation

**Published:** 1859-02

**Authors:** 


### NATURE AND REVELATION.

The God of both is one and the same. In the operations of both the same great general principles run parallel. In the vegetable world, the world of mind and the world of grace, there are the same great changes of seed time and harvest—of ebb and flow—of renewal and decay—of increment and loss—of opportunity improved or forfeited—of chances used, or for ever gone.

Every spring the vegetable world takes a new lease of life; every morning man wakes up to renewed vigor. In the human body, too, there are times which, more than any others, are adapted to the renovation of health and to the arrest of sickness; but, if unimproved, the vigor of manhood declines, disease burrows in the system, and there is no repair. Nor is it different in the momentous world of grace. Ordinarily a man may at any time become a Christian; but there are seasons of extraordinary fructification, when the facilities are so largely increased, that resistance, refusal to employ them, is a madness, a fatuity; because, if rejected then, the offer may be made no more. It is certainly true in the life of every man that there are critical periods, which, if rightly improved, add many years to his age. These periods regularly recur, and, if *not* improved, that man never lives to see another. The fructifying shower does not always fall, and the sheltered plant, which needed it so much, will die long before another comes. And just as certain is it in this time of "great awakenings," that multitudes who stand under the spiritual showers but ward them off by feelings of indifference, or shame, or greed of gold, or thirst for human applause, or love of festivity, revelry, and mirth, or the fatal indecision, which is the

> "Thief of time!
> Year after year it steals, till all are fled,
> And to the mercies of a moment leaves
> The vast concerns of an immortal scene."

To doubt or under-estimate these special opportunities, because they are unusual, or transient, or may fail of permanent benefit to some, is to be like a simpleton gardener, who protects his plants against the shower because it falls at an unusual season, or because it is not sufficient, in his estimation, to produce any other than a temporary good effect, except to a portion of them; or like the unthinking invalid, who, racked with torture, refuses to take the soothing medicament because its good effects may soon pass away. So also are there times more than ordinarily propitious for the securement of health and the prompt arrest of the advance of insidious disease. Youth is the time for the former, as also about the age of forty years. As to the latter, "prompt attention" is the universal rule, given at length in our new book, "Health and Disease."

